# Activated cMET and IGF1R-Driven PI3K Signaling Predicts Poor Survival in Colorectal Cancers Independent of KRAS Mutational Status

**DOI:** 10.1371/journal.pone.0103551

**Published:** 2014-08-04

**Authors:** Jeeyun Lee, Anjali Jain, Phillip Kim, Tani Lee, Anne Kuller, Fred Princen, In-Gu, Suk Hyeong Kim, Joon Oh Park, Young Suk Park, Sharat Singh, Hee Cheol Kim

**Affiliations:** 1 Division of Hematology-Oncology, Department of Medicine, Samsung Medical Center, Sungkyunkwan University School of Medicine, Seoul, Korea; 2 Departments of Surgery, Samsung Medical Center, Sungkyunkwan University School of Medicine, Seoul, Korea; 3 Research and Development, Oncology, Prometheus Laboratories, San Diego, California, United States of America; 4 Pathology, Samsung Medical Center, Sungkyunkwan University School of Medicine, Seoul, Korea; Sapporo Medical University, Japan

## Abstract

**Background:**

Oncogenic mutational analysis provides predictive guidance for therapeutics such as anti-EGFR antibodies, but it is successful only for a subset of colorectal cancer (CRC) patients.

**Method:**

**A** comprehensive molecular profiling of 120 CRC patients, including 116 primary, 15 liver metastasis, and 1 peritoneal seeding tissue samples was performed to identify the relationship between v-Ki-ras2 Kirsten rat sarcoma viral oncogene homolog (*KRAS*) WT and mutant CRC tumors and clinical outcomes. This included determination of the protein activation patterns of human epidermal receptor 1 (HER1), HER2, HER3, c-MET, insulin-like growth factor 1 receptor (IGF1R), phosphatidylinositide 3-kinase (PI3K), Src homology 2 domain containing (Shc), protein kinase B (AKT), and extracellular signal-regulated kinase (ERK) kinases using multiplexed collaborative enzyme enhanced reactive (CEER) immunoassay.

**Results:**

*KRAS* WT and mutated CRCs were not different with respect to the expression of the various signaling molecules. Poor prognosis in terms of early relapse (<2 years) and shorter disease-free survival (DFS) correlated with enhanced activation of PI3K signaling relative to the HER kinase pathway signaling, but not with the *KRAS* mutational status. *KRAS* WT CRCs were identified as a mixed prognosis population depending on their level of PI3K signaling. *KRAS* WT CRCs with high HER1/c-MET index ratio demonstrated a better DFS post-surgery. c-MET and IGF1R activities relative to HER axis activity were considerably higher in early relapse CRCs, suggesting a role for these alternative receptor tyrosine kinases (RTKs) in driving high PI3K signaling.

**Conclusions:**

The presented data subclassified CRCs based on their activated signaling pathways and identify a role for c-MET and IGF1R-driven PI3K signaling in CRCs, which is superior to KRAS mutational tests alone. The results from this study can be utilized to identify aggressive CRCs, explain failure of currently approved therapeutics in specific CRC subsets, and, most importantly, generate hypotheses for pathway-guided therapeutic strategies that can be tested clinically.

## Introduction

Monoclonal antibodies such as cetuximab and panitumumab that target the epidermal growth factor receptor (EGFR), a human epidermal receptor (HER) family member, have proven to be efficacious in terms of response rate and progression-free survival in combination with standard cytotoxic chemotherapy in metastatic colorectal cancers (CRCs) [1]–[4]. The EGFR targeting antibodies bind to the extracellular domain of EGFR, leading to the inhibition of its downstream signaling pathways, including the RAS-RAF-mitogen-activated protein kinase 1 (MAPK1) axis that is mainly involved in cell proliferation, and the v-akt murine thymoma viral oncogene homolog 1 (AKT1) pathway, which is mainly involved in cell survival and tumor invasion [5]. AKT1 is regulated by the upstream phosphatidylinositol 3-kinase (PI3K) signaling pathway.

Mutations in the v-Ki-ras2 Kirsten rat sarcoma viral oncogene homolog (*KRAS*), most frequently detected in codons 12, 13, and 61, occur in approximately 40% of CRC patients [6], [7]. *KRAS* mutations have emerged as the key negative predictive factor for treatment response in patients receiving cetuximab [8], [9]. These studies have suggested that *KRAS* wild-type (WT) CRC tumors would be responsive to cetuximab; however, up to 65% of patients with *KRAS* WT tumors are still resistant to anti-EGFR monoclonal antibodies [10]. Resistance to anti-EGFR antibodies in a subset of *KRAS* WT CRCs can be explained by the presence of a mutation within the *BRAF* oncogene [8], which is downstream of *KRAS*. The reason for cetuximab non-response in the remaining *KRAS* WT CRCs remains unclear. Furthermore, although *KRAS* mutations are typically associated with non-responsiveness to anti-EGFR antibodies, recent data indicate that *KRAS* G13D mutations may be a positive predictor of cetuximab response [8]. Mutations within the *PIK3CA* gene [10], which is an important regulator of PI3K signaling, are also present in some CRC tumors that can co-occur with *KRAS* or *BRAF* mutations [8], [11], thus suggesting their possible influence on responsiveness to targeted therapeutics such as anti-EGFR antibodies but a clear demonstration of such a correlation is lacking [12], [13].

From the studies outlined above and given that a large proportion of CRC patients with *KRAS* WT tumors do not respond to cetuximab or panitumumab, it is clear that a simple mutational analysis is insufficient to predict responsiveness to such therapeutics. In addition, since recent studies suggested that the therapeutic responses to PI3K inhibitors were not limited to colorectal cell lines with activating mutations or in patients with mutations [14]–[16], it is imperative to profile tumors for their predominant as well as potential alternate signaling pathway drivers in addition to the oncogenic mutational analysis. Therefore, we aimed to profile CRC tissues to investigate the correlation between mutational status and various receptor tyrosine kinase (RTK) protein expressions such as HER1, HER2, HER3, c-MET, and insulin-like growth factor 1 receptor (IGF1R). In addition, downstream kinases PI3K, Src homology 2 domain containing (Shc), protein kinase B (AKT), and extracellular signal-regulated kinase (ERK) were determined using the multiplexed immunomicroarray based Collaborative Enzyme Enhanced Reactive (CEER) immunoassay [17]–[19] in 120 CRC patients from stage I to IV that included 116 primary, 15 liver metastasis, and 1 peritoneal seeding tissue samples. In parallel, somatic mutational analysis scored for mutations within the *KRAS* and *BRAF* oncogenes. CRC tumors with similar oncogenic mutations demonstrated heterogeneity in their signaling pathway profiles.

## Patients and Methods

### Patient Cohort and Tissue Specimen Procurement

The study was approved by the institutional review board (IRB) at Samsung Medical Center. All clinical investigation was conducted according to the principles expressed in the Declaration of Helsinki. The written informed consent was waived by the IRB due to retrospective analysis and anonymous data. Fresh frozen tissues (n = 120) collected from surgically resected tumors (73 colon, 47 rectum), 15 from metastatic liver tumors and one from peritoneal seeding nodule, were available for final analysis. All fresh frozen tissues were collected within 30 min at the surgical field by a surgeon, were immediately snap-frozen in liquid nitrogen, and stored at −80°C until use. Tumor specimens were confirmed for the presence of tumor >70% area by a pathologist. For the analysis, small pieces of frozen tissues (10-µm sections ×3) were prepared using prechilled razor blades and were then lysed in 100 µL of lysis buffer. The resulting lysates were stored at −80°C until subsequent analysis.

### Collaborative Enzyme Enhanced Reactive-Immunoassay (CEER)

CEER uses an antibody microarray-based platform that can measure the expression and activation levels of signal transduction proteins in tumor tissues and surrogate tissues. The selected target is first captured by a target-specific capture antibody followed by co-localization of two additional detector antibodies against the same target, eventually resulting in specific target detection and quantitation. Detailed methods for this technology have been described previously [17]–[19] and can be found in the File S2. Representative experiments are shown in Fig.S1 in File S1.

### Mutation analysis

Genomic DNA was extracted from CRC tissues using the Qiagen Tissue Kit. Samples were screened for mutations in *KRAS*, *BRAF*, and *PIK3CA* genes: G12A/C/D/S/V, G13C/D, and Q61H in *KRAS* and V600E in *BRAF*. The mutational assay was based on the TaqMan real time polymerase chain reaction (PCR) technology in combination with allele specific primers (ASP), blocker, and probe using a modification of the real-time Allele Specific PCR detection method [20]. Briefly, ASPs were used to specifically detect the mutant allele. A blocking oligonucleotide (blocker) complementary to the wild-type sequence was used to suppress any non-specific amplification of the wild-type allele. The PCR mix used for all the assays was GTXpress Master Mix from Life Technologies. All the assays were run on 384-well plates ABI 7900HT Real Time PCR Instrument (Life Technologies).

The percentage of the allelic variant present in unknown samples was calculated using a standard curve. The standard curve was generated for each mutation from the DNA extracted from a cell line positive for that mutation using a series of DNA dilutions (100, 10, 1, 0.1, and 0.01 ng). A list of the positive cell lines used for generating the standard curves is shown in the table below. The DNA from the respective cell lines was extracted using the Qiagen DNeasy Blood & Tissue Kit.

Using the standard curve, the amount of the respective mutation in the unknown DNA sample was calculated from the Ct value that could then be used to calculate the percent allelic variant based on the assumption that the standard cell line is 100% positive for that specific mutation. The allelic variance of the cell lines was determined using primers specific to the wild-type allele. Following are the cell lines used for gene mutations: SW1116(KRASG12A), NCI-H23(KRASG12C), LS174T (KRASG12D), PSN1(KRASG12R), A549(KRASG12S), SW403(G12V), H1734(KRASG13C), T84(KRASG13D), H460(KRASQ61H), and HT29(BRAFV600E).

### Statistical analysis

Mann-Whitney *t*-tests, Kaplan-Meier survival analysis, and Pearson correlation analysis were performed using GraphPad Prism version 5 for Mac OS X (GraphPad Software, La Jolla California USA, www.graphpad.com). DFS was determined using the Kaplan–Meier method, and survival curves were compared using the Log-ratio method. Survival was measured from the date of surgery. All tests were two-sided, and P values less than 0.05 were considered significant. Statistical analysis was performed using SPSS 20 software for Windows (SPSS Inc., Chicago, IL).

## Results

### Patient Characteristics

The characteristics of the 120 CRC patients are provided in Table 1. Seventy-three patients presented with a colon primary, whereas 47 patients had rectum primary. Approximately 70% of the patients underwent adjuvant chemo- or radiotherapy depending on the primary tumor location. Ten colon-liver metastasis pairs and one colon-peritoneal seeding pair were included in the analysis. All tissues, including paired specimens, were procured at the time of surgery and immediately snap-frozen at the surgical field for future molecular analysis.

**Table 1:** Patient characteristics.

Characteristics	(Total N = 120) (%)
Age	
Median age (range)	61 (31–85)
≤65	68 (56.7)
>65	52 (43.3)
Sex	
Male	73 (60.8)
Female	47 (39.2)
Stage	
I	11 (9.2)
II	38 (31.7)
III	38 (31.7)
IV	33 (27.4)
Histology	
WD	8 (6.7)
MD	99 (82.5)
PD	5 (4.1)
Adenocarcinoma, not specified	8 (6.7)
Primary tumor	
Colon	73 (60.8)
Rectum	47 (39.2)
Curative resection	
Yes	108 (90.0)
No	12 (10.0)
Adjuvant chemotherapy (Colon)	
5-FU based chemotherapy	20 (27.4)
Capecitabine	18 (24.7)
XELOX or XELIRI	13 (17.8)
No adjuvant chemotherapy	22 (30.1)
Adjuvant chemotherapy (Rectum)	
5-FU+RT	28 (59.6)
capecitabine/oxaliplatin+RT	1 (2.1)
5-FU based chemotherapy	2 (4.3)
capecitabine	1 (2.1)
No adjuvant chemotherapy	15 (31.9)
Tissue specimens (from 120 patients)	
Primary (colon or rectum)	116
Liver metastasis	14 (10 paired with primary)
Peritoneal seeding	1 (paired with primary)
KRAS mutational status	
At least one mutation	49 (42%)
KRAS G12S	3
KRAS G12D	19
KRAS G12A	1
KRAS G12V	6
KRAS G12C	7
KRAS G13D	17

The characteristics of the 120 patients used in this study are summarized.

WD: Well differentiated; MD: Moderately differentiated; PD: Poorly differentiated.

### Differential signaling pathway activations predicted poor survival better than KRAS mutations

**Figure 1:**
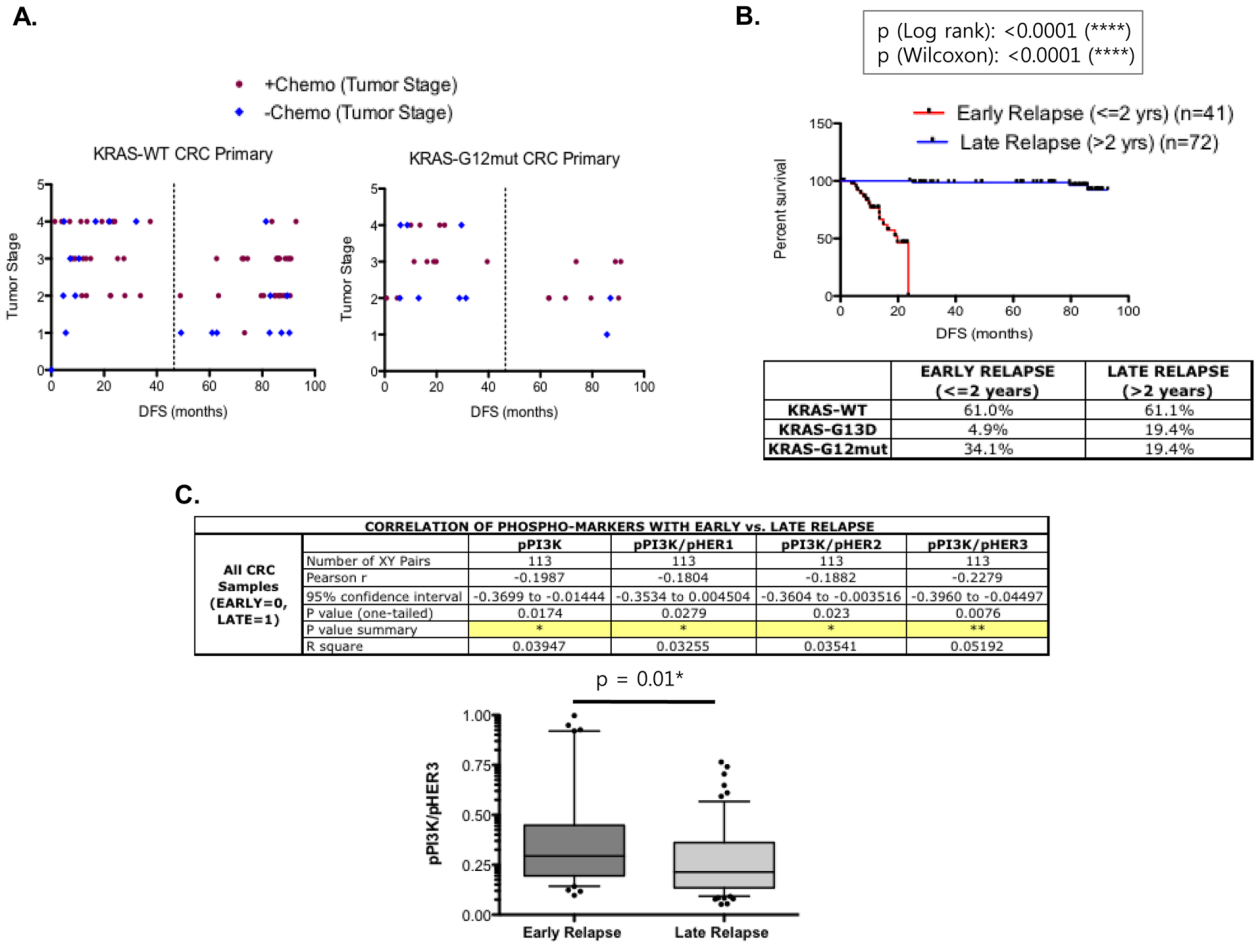
Heterogeneity in colorectal cancers (CRCs). (A) Plot of disease-free survival (DFS) versus tumor stage in *KRAS* WT and G12mut primary CRCs. Each dot represents an individual patient and the brown and blue dots indicate patients that did or did not receive adjuvant chemotherapy, respectively. (B) Comparative DFS curves of CRC samples segregated by their recurrence times post-surgery. DFS for early relapse patients (recurrence ≤2 years) is shown in red and DFS for late relapse patients (recurrence >2 years) is shown in blue. p-Values are indicated. The table below the graph lists the percentage of patients with a specific *KRAS* genotype in each group. (C) Pearson correlation analysis of early vs. late relapse CRCs to the expression of pPI3K, pPI3K/pHER1, pPI3K/pHER2, and pPI3K/pHER3 in the entire CRC cohort. Significant correlations are highlighted in yellow. The plot in the box shows the difference in the pPI3K/pHER3 ratios in early relapse vs. late relapse CRCs.

*KRAS* mutations, with *KRAS* G12D and G13D mutations being the most frequent, were observed in 39% (45/115) of the samples in our CRC patient cohort. In particular, 28/115 patients carried the *KRAS* G12 mutations and 17/115 patients carried the *KRAS* G13D mutations. Mutations in *BRAF*, which is downstream of *KRAS*, were found in 4% (5/115) of the patients. Although *KRAS* G12-mutated CRCs are generally associated with poor prognosis, a considerable percentage of *KRAS* WT CRCs showed low DFS and some *KRAS* G12 mutated CRCs showed high DFS regardless of their tumor stage (Fig. 1A). Tumors in each *KRAS* mutational subtype demonstrated a similar median expression of phosphorylated ERK (pERK), which is downstream of *KRAS* (Fig. S1). An equivalent percentage of CRC tumors expressed pERK above the median (51.8% in *KRAS* WT, 48.5% in G12 mutated, and 44.4% in G13D mutated tumors).

As expected, the survival curves for the two cohorts were significantly different (Fig. 1B), with the respective median survival times of 19.8 months for early relapse (2 years from surgery) and undefined for late relapse (hazard ratio = 117.5). As shown in Fig. 1C, higher expression of activated PI3K correlated with early relapse and specifically, higher expression of pPI3K/pHER ratios was significantly associated with an early relapse. PI3K is an important downstream signaling effector of the HER kinase axis with direct binding sites on HER3. The cut off value for pPIK3/HER3 ratio was determined as shown in Fig.S2 in File S1. Furthermore, pPI3K/pHER3 expression was significantly higher in CRC tumors that relapsed within 2 years (Fig. 1C). Therefore, enhanced PI3K signaling was observed in CRCs with a shorter DFS or poorer prognosis.

### High PI3K signaling is associated with early recurrence in CRC

**Figure 2:**
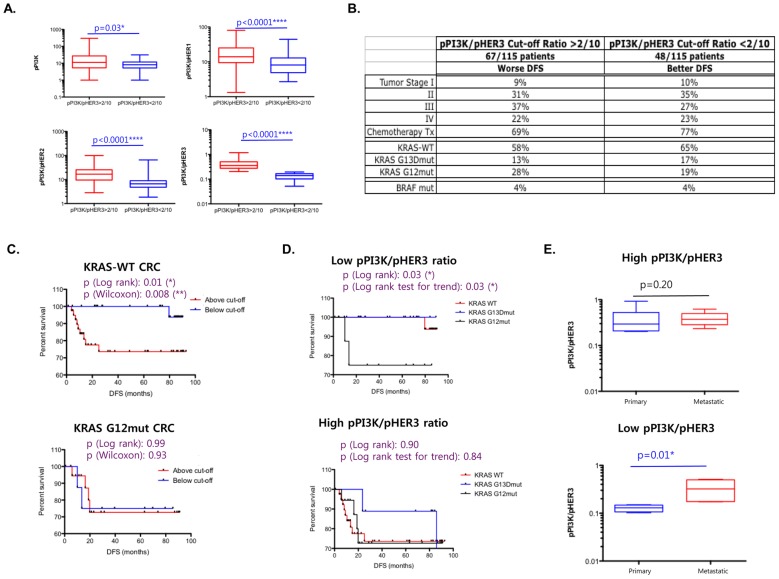
PI3K signaling in colorectal cancers (CRCs). The respective p-values are indicated. (A) Box plots of the Mann–Whitney *t*-test showing the difference in the expression of pPI3K, pPI3K/pHER1, pPI3K/pHER2, and pPI3K/pHER3 between the high pPI3K/pHER3 group and low pPI3K/pHER3 groups. The respective p-values are indicated. (B) Table listing the characteristics of primary CRCs in high PI3K (high pPI3K/pHER3) versus low PI3K (low pPI3K/pHER3) signaling cohorts. Characteristics include segregations based on tumor stage, status of chemotherapy treatment, and mutational status of *KRAS* and *BRAF*. Altogether, 67/115 samples are included in the high PI3K cohort and 48/115 samples are included in the low PI3K cohort. The numbers indicate the percentage of samples within each cohort based on each indicated characteristic. (C) DFS according to pPI3K/pHER3 ratios in *KRAS* WT and *KRAS* G12mut CRCs. (D) Kaplan–Meier survival curves of high PI3K (pPI3K/pHER3) and low PI3K cohorts comparing the DFS of *KRAS* WT, G13Dmut, and G12mut samples. (E) high PI3K (high pPI3K/pHER3) and low PI3K (low pPI3K/pHER3) groups in paired primary and metastatic CRC samples.

To gain further insight into the high vs. low pPI3K signaling CRC cohorts, we focused on the CRC sub-cohorts segregated by the pPI3K/pHER3 ratio (Fig. 2A). Patient characteristics in the high vs. low pPI3K signaling CRC cohorts were well balanced in terms of their tumor stage, chemotherapy treatments, and mutational status of the oncogenes *KRAS* and *BRAF* (Fig. 2B). DFS of *KRAS* WT CRCs with higher pPI3K/pHER3 expression was significantly worse than that of *KRAS* WT CRCs with lower pPI3K/pHER3 expression (Fig. 2C); however, *KRAS* G12mut tumors demonstrated poor DFS regardless of their level of pPI3K expression. Interestingly, DFS comparisons of *KRAS* WT, G13Dmut, and G12mut CRCs within the low pPI3K group showed considerable differences, with *KRAS* WT and *KRAS* G13D tumors having a better survival than the *KRAS* G12mut tumors (Fig. 2D). Despite the dramatic differences in survival observed in the high and low pPI3K/pHER3 groups depending on their *KRAS* WT or G12 mutated genotype, the two groups were very similar in terms of their expression differences of the relevant markers. In other words, pPI3K/pHER1, pPI3K/pHER2, and pPI3K/pHER3 were expressed to considerably higher levels in the high pPI3K/pHER3 group in both *KRAS* WT and G12 mutated CRCs (Fig. S3 in File S1). Note that the pPI3K expression was not considerably higher, but it was the pPI3K expression relative to the activated HER axis that was higher in the groups showing poor survival.

These data provided evidence that a high pPI3K signaling, which may possibly be driven by upstream signals other than or in addition to the HER axis, is associated with aggressive CRCs with early recurrence. Furthermore, these data clearly demonstrated the heterogeneity within the *KRAS* WT population based on the level of pPI3K expression in these tumors. On the other hand, *KRAS* G12-mutated CRCs typically demonstrated poor survival regardless of the level of pPI3K signaling.

### PI3K-driven aggressive CRCs, including metastatic CRCs, show higher c-MET and IGF1R signaling than the HER axis signaling

Since higher pPI3K/pHER ratios correlated with early relapse CRCs, we hypothesized that there should also be a significant difference in pHER/pMET and pHER/pIGF1R ratios if pMET and pIGF1R are responsible for high pPI3K signaling. Next, we examined these signaling networks in 10 pairs of matched primary-metastatic synchronous samples available in our study cohort. The primary-metastatic CRC pairs were segregated into two groups based on the pPI3K/pHER3 expression ratio of the primary CRC sample in each pair. There was a significant increase in the pPI3K/pHER3 ratio for the matched metastatic samples in the low pPI3K/pHER3 ratio group (Fig. 2E), whereas it remained unchanged between primary and metastatic samples in the high pPI3K/pHER3 ratio group.

### Aggressiveness of KRAS G12mut CRCs is correlated with high c-MET expression relative to HER axis members

**Figure 3:**
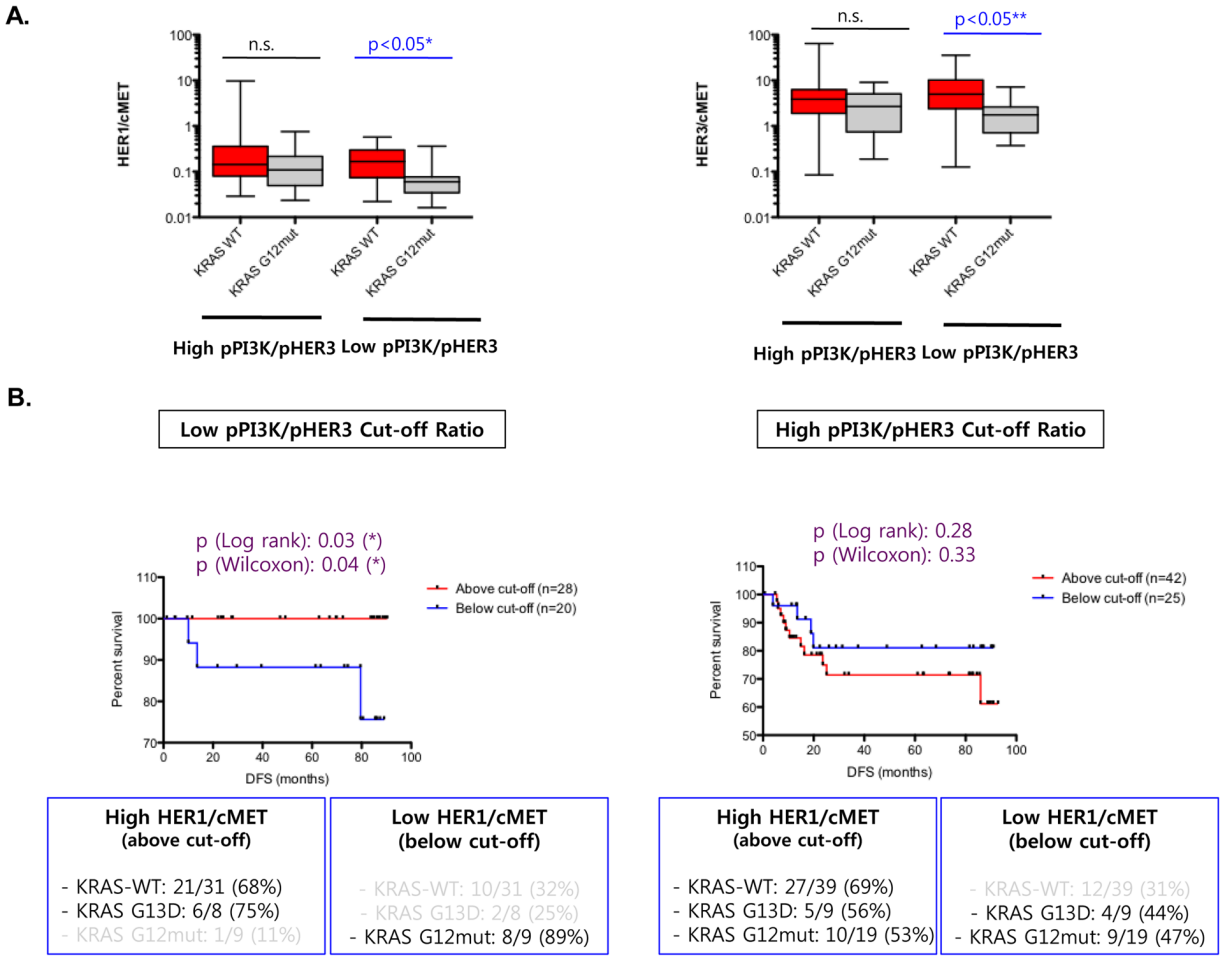
Relative c-MET and IGF1R expression in high and low PI3K signaling colorectal cancer (CRC) cohorts. (A) pMET/T-c-MET ratio was compared between high PI3K (high pPI3K/pHER3) and low PI3K (high pPI3K/pHER3) groups using the Mann–Whitney *t*-test. The comparison is shown in all CRCs, *KRAS* WT, G13D mutated, and G12 mutated CRCs. Significant differences with p-values are indicated in blue. (B) Comparative expression between high and low PI3K groups for the following markers: pHER1/pMET, pHER2/pMET, pHER3/pMET, pHER1/pIGF1R, pHER2/pIGF1R, and pHER3/pIGF1R. Significant differences with p-values are indicated in blue.

We then attempted to identify markers that may be differentially expressed between *KRAS* WT and G12mut CRCs in the low pPI3K group. Based on our preliminary observation of high expression of total c-MET and IGF1R in *KRAS* G12mut tumors compared to the *KRAS* WT tumors (Fig. 3A, Fig. S4 in File S1), we examined whether a differential c-MET- or IGF1R-dependent expression may segregate the *KRAS* WT and G12mut subgroups within the low pPI3K/pHER3 ratio group. Relative HER1/c-MET and HER3/c-MET ratios were considerably higher in the *KRAS* WT CRCs than in *KRAS* G12mut tumors (Fig. 3B) in the low pPI3K/pHER3 group, but not in the high pPI3K/pHER3 group, which was consistent with the DFS differences (Fig. 3A). We investigated whether appropriate ratios of HER1/c-MET (Fig. S5 in File S1) or HER3/c-MET may also segregate the DFS differences observed between the *KRAS* WT and G12mut CRCs within the low pPI3K/pHER3 ratio group. Analysis of the *KRAS* genotypes of the samples in the two sub-cohorts revealed that the majority of the *KRAS* WT and G13D samples were present in the high HER1/c-MET sub-cohort (i.e., HER1 > c-MET), whereas the majority of the *KRAS* G12mut samples were present in the low HER1/c-MET sub-cohort (i.e., HER1 < c-MET) (Fig. 3C). A similar HER1/c-MET cut-off ratio did not segregate the high pPI3K/pHER3 group based on the *KRAS* genotypes (Fig. 3D). Parallel analysis with a HER3/c-MET index resulted in similar but less robust data that did not segregate the KRAS WT and G12mut CRCs as distinctly as the HER1/c-MET index (*data not shown*). These data indicated that the aggressive *KRAS* G12mut tumors are molecularly distinct because they are mostly marked by a high c-MET expression, which is equivalent to or higher than HER1 and HER3 expression.

## Discussion

**Figure 4:**
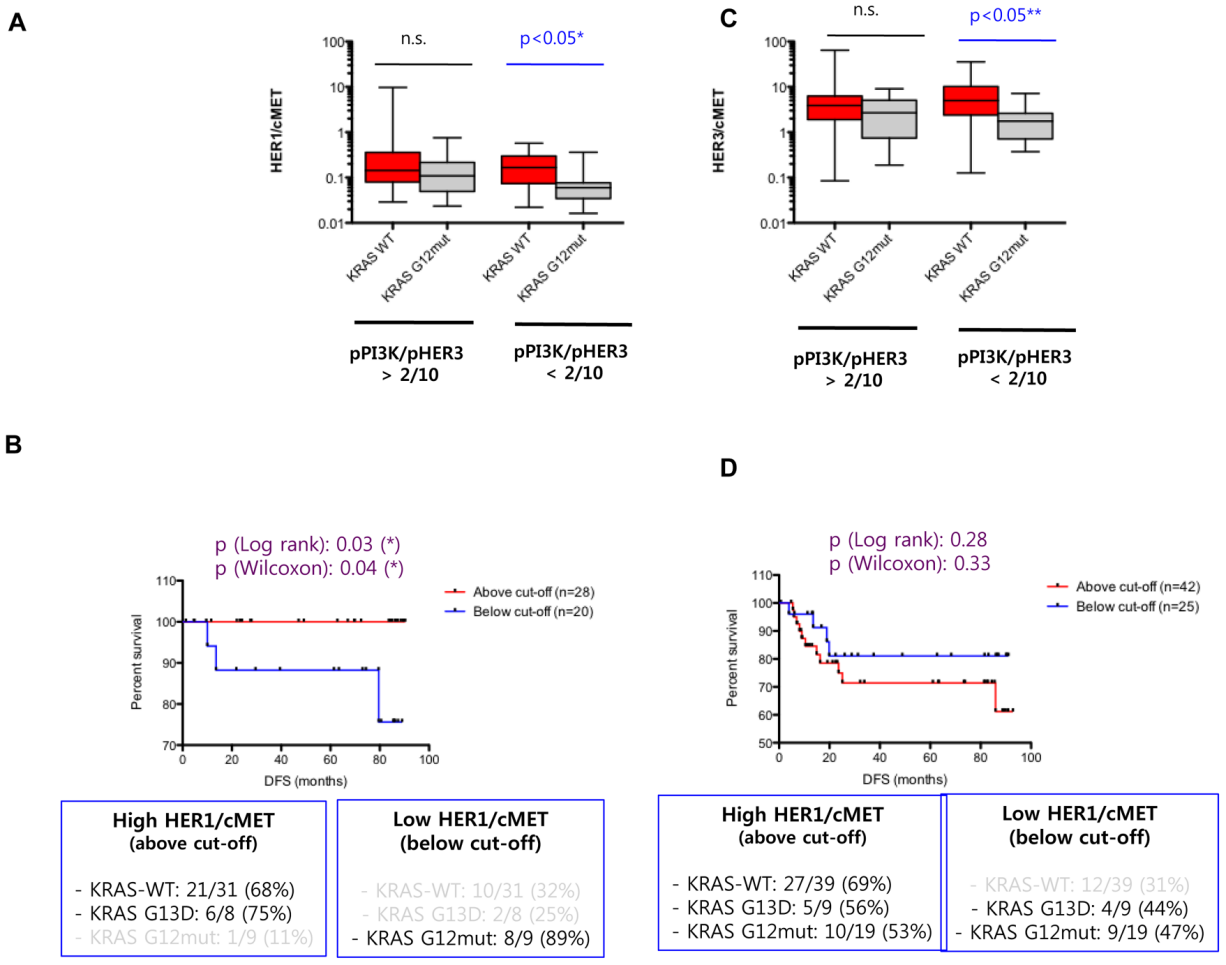
Summary of colorectal cancer (CRC) sub-classifications based on their signaling pathway profiles. Schematic summarizing the sub-classification of CRCs based on their signaling pathway profiles and *KRAS* mutational status. The possible therapeutic options for each sub-class based on their pathway profiles are indicated.

This study was based on the hypothesis that the status of activated signaling pathways in *KRAS* WT and mutant CRCs can provide additional information that may be useful for understanding the therapeutic outcomes in these tumors [17]–[19]. The analysis presented in this study gives evidence that CRC can be sub-classified based on its signaling pathway molecular profiles regardless of the *KRAS* mutational status, as summarized in Fig. 4. Besides providing molecular insights into CRC prognosis, such a pathway-based profiling can have important implications on stratifying the CRC patient population for appropriate therapeutic strategies.

Prognosis of CRC primary tumors post-surgery has been correlated with their *KRAS* mutational status [21]. Although this general trend was also observed in our CRC sample cohort, with *KRAS* WT tumors demonstrating better DFS post-surgery than *KRAS* G12 mutant tumors, it was not significant due to the heterogeneity in the signaling pathways within each *KRAS* mutational subtype, especially in stage II and III CRC tumors regardless of whether or not the patients received chemotherapy. This suggests that *KRAS* mutations confer only part of the advantage needed for tumor cell survival with additional survival signals presumably deriving from multiple signaling pathways. CRCs with high PI3K activity relapsed within 2 years independent of their *KRAS* mutational status and had poor prognosis. Even *KRAS* WT CRCs with high PI3K signaling were as aggressive as the *KRAS* G12mut CRCs with indistinguishable DFS post-surgery. In contrast, *KRAS* WT CRCs with low PI3K signaling activity demonstrated a better prognosis and could be segregated from the aggressive *KRAS* G12mut CRCs with an appropriate HER1/c-MET index cut-off ratio, because c-MET expression levels were higher in *KRAS* G12mut CRCs. In our study cohort, these *KRAS* WT CRCs constituted ∼44% of the total *KRAS* WT population and revealed heterogeneity. Heterogeneity in *KRAS* WT CRC populations has been previously noticed in numerous other studies, because not all *KRAS* WT CRCs are responsive to anti-EGFR antibodies. One possible reason has been described as the presence of *BRAF* mutations. The number of *BRAF*-mutated samples in our cohort was too small to draw any reasonable conclusions; however, 3/5 of *BRAF*-mutated CRCs in the presence of WT *KRAS* demonstrated a high PI3K signaling. Our study provides a possible mechanism for the heterogeneity in *KRAS* WT CRCs and we speculate that 44% of the *KRAS* WT CRCs with low PI3K signaling and high HER1 expression may be those responsive to anti-HER therapies such as anti-EGFR antibodies. Approximately 47% of the CRC samples with *KRAS* G13D mutations were recently suggested to have distinct therapy-based outcome characteristics [8] and were tracked with our *KRAS* WT samples in terms of having low PI3K signaling and a high HER1 expression. The relevance of PI3K signaling and PI3K/mTOR inhibitors has been previously suggested in *KRAS* mutant [22] and *BRAF* mutant [23] CRCs. The results from our study are consistent with these reports and further expand the importance of PI3K signaling in CRCs regardless of their mutational status.

Relatively high c-MET and IGF1R activities compared to HER kinase members activities in aggressive CRCs suggested that these alternative RTKs may be the drivers of the high PI3K signaling. *KRAS* G12-mutated CRCs were noted to express higher c-MET receptor levels than the HER members that correlated with their poor prognosis. Most compelling evidence in support of c-MET- and IGF1R-driven PI3K activity in aggressive CRCs came from the primary-metastatic sample pairs, in which these markers showed a clear increase in the metastatic counterpart when compared to the matched primary sample, even though the mutational status of the pair remained unchanged. It is important to note that expression differences for single markers did not present meaningful differences but it was the relative expression of pHER signaling to pMET and pIGF1R that revealed considerably differential patterns between CRCs that were more aggressive and relapsed earlier versus the ones that were associated with better prognosis. Currently, there are no tangible options to identify aggressive CRCs and defining therapeutic options to treat them. Mutational tests are the only available strategy, which does not identify the aggressive *KRAS* WT CRCs. Our study provides evidence for activated signaling pathway profiles that are superior to oncogenic mutational tests alone, because these results can be utilized to identify aggressive CRCs and possibly guide therapeutic strategies. The clinical implication of combinatorial protein profiling and mutational tests in terms of drug sensitivity is currently being tested both in preclinical and prospective clinical studies.

Our study also uncovered a substantial variation in phosphorylated proteomic profiling between matched primary and metastatic CRCs. This was in contrast with genotyping, according to which there was complete concordance between primary and metastatic tumor tissues. This is in line with the results from one of the largest comparison studies between primary and matched liver metastases in 305 CRC patients (concordance rate, 96.4%; 95% confidence interval, 93.6–98.2%) [24]. Furthermore, a recent genomic analysis on 84 patients with primary CRC and liver metastases demonstrated a high concordance rate of >90% between primary and liver metastases for five genes (i.e., *KRAS*, *NRAS*, *BRAF*, *PIK3CA*, *TP53*) [25]. One of the most plausible hypotheses for such high concordance rate in the mutational status is that *KRAS* mutations are the early driving events in CRC progression from adenoma [26]. If these differences in pathway profiling correlate with the treatment responses to specific RTK inhibitors, we may need to biopsy metastatic sites in addition to primary tumors to obtain a more precise prediction of the treatment response. However, our sample size was very small with only ten pairs of primary and metastasis. Hence, more extensive paired analysis is needed to rigorously address this discrepancy in proteomic profiling.

In conclusion, our study demonstrates that there is significant heterogeneity in activated protein signaling pathways despite a similar mutational status in CRC. Therefore, dichotomizing CRC simply as *KRAS* mutant versus *KRAS* WT may be an underestimation of the molecular heterogeneity within each subgroup of CRC. Moreover, a more comprehensive signaling pathway profiling, in addition to oncogenic mutational tests, should be performed to obtain a clearer molecular identity of the tumors.

## Supporting Information

File S1 **Supporting Figures. Figure S1. Profiling of signaling pathway markers in colorectal cancers using CEER.** (A) Ranking of pERK expression from high to low in *KRAS* wild-type, G12 mutated and G13D mutated CRCs is shown in a waterfall plot. pERK expression above the median is represented on the positive y-axis. pAKT expression in each sample is also shown. The table below shows the median pERK CU values in each mutational subgroup of CRC tumors as well as the percentage of tumors that express pERK above the median. (B) CEER immuno-array images for indicated signal transduction proteins in 8 colorectal samples. As represented with a color bar below the immuno-array, a white or a red represents a high level expression or phosphorylation whereas a green or a blue represents a low level expression or phosphorylation of the respective markers. Tumor stage and the *KRAS* mutational status of each sample are indicated. **Figure S2. Effect of pPI3K/pHER3 index on disease-free survival in CRCs.** Comparative DFS curves of CRC samples segregated by decreasing pPI3K/pHER3 ratios. DFS for samples above each respective cut-off is shown in red and DFS for samples below each respective cut-off is shown in blue. Respective p-values are indicated. **Figure S3. Characteristics of matched primary-metastatic CRC sample pairs.** Table listing primary tumor characteristics of the 10 matched primary-metastatic CRC pairs segregated by their pPI3K/pHER3 expression ratios. 6 pairs of matched samples are included in the worse DFS group where pPI3K/pHER3 expression was >2/10. Characteristics include number of samples in each tumor stage, whether the patient received chemotherapy and number of samples based on the mutational status of *KRAS*, *BRAF* and *PIK3CA*. **Figure S4. Comparative signaling marker expression between** ***KRAS*** **WT and G12 mutated CRCs.** Box plots showing Mann Whitney t-test based expression differences in total and phosphorylated HER1, HER2, HER3, cMET, IGF1R, pPI3K, pSHC, pAKT and pERK in KRAS WT and G12 mutated CRCs. Significant differences with respective p values are indicated. **Figure S5. DFS segregation based on HER1/cMET index.** Comparative DFS curves of CRC samples within the pPI3K/pHER3<2/10 group segregated by decreasing HER1/cMET ratios. DFS for samples above each respective cut-off is shown in red and DFS for samples below each respective cut-off is shown in blue. Respective p-values are indicated.
(PPTX)

File S2 **Supplementary Methods.**
(DOCX)
