# Supplementary material for: Activated cMET and IGF1R-Driven PI3K Signaling Predicts Poor Survival in Colorectal Cancers Independent of KRAS Mutational Status
Source: PLoS One. 2014 Aug 4;9(8):e103551. doi: 10.1371/journal.pone.0103551 (PMC4121133; doi:10.1371/journal.pone.0103551)
Supplement: File S2 — Supplementary Methods. (DOCX) [file pone.0103551.s002.docx]

**Supplementary Methods**

***Collaborative Enzyme Enhanced Reactive-immunoassay (CEER)***

Capture antibodies were printed on nitrocellulose-coated glass slides (ONCYTE®, Grace Bio-Labs) using non-contact printers (Nanoplotter, GeSiM). The spot diameter was approximately 175 µm, slides were kept in a desiccated chamber. Approximately 500 pL of capture Abs were printed in triplicate and serial dilution concentrations of 1 mg/ mL, 0.5mg/ mL, and 0.25mg/ mL. Purified mouse-IgGs served as negative controls. Immuno-array slide configurations and assay format was described previously [[17-19](#_ENREF_17)].

Immuno-microarray slides were rinsed 2x with TBST (50 mM Tris/ 150 mM NaCl/ 0.1% Tween-20, pH 7.2-7.4), blocked with 80 µL Whatman Blocking Buffer 1hr at RT, then washed 2x with TBST. Serially diluted lysate controls in 80 µL dilution buffer (2% BSA/ 0.1% TritonX-100/ TBS, pH 7.2-7.4) and samples were added to designated sub-arrays on slides, then incubated 1 hour at RT. Slides were washed 4x (3 min. each), and detector Abs were added in 80 µL of reaction buffer and incubated for 2 hours at RT. After washing slides with TBST to remove unbound detector Abs, 80 µL of biotin-tyramide solution (5 µg/ml in 50 mM glucose/PBS) prepared from 400 µg/ mL in ethanol solution (Perkin-Elmer Life Science) was added and incubated 15 min in darkness. Glucose-oxidase (GO)/HRP-mediated tyramide signal amplification process was terminated by washing with TBST 4x, 3 min each. Local deposition of biotin-tyramide was detected by incubation with streptavidin (SA)-Alexa647 (Invitrogen) at 0.5 µg/mL in 2% BSA/ 0.1% Triton/TBS for 40 min. Upon completion of incubation, slides were washed 4x with TBST, dried and kept in darkness until imaged via microarray scanner.

Each slide was scanned at four photomultiplier (PMT) gain settings to increase the effective dynamic range. Background corrected signal intensities were averaged for replicate spots printed in triplicate. The relative fluorescence value of the respective reagent blank was subtracted from each sample. Several quality criteria were used to filter data from further analysis including limits on the spot footprint, coefficient of variation for spot replicates, overall pad background and the intensity of the reagent blank.

For each assay, a sigmoidal standard curve was generated from seven concentrations of serially diluted cell lysates prepared from cell lines (MDA-MB-468 (HER1 positive), BT474 (HER2 positive), T47D (HER3 and IGF1R positive), HCC827 (cMET positive) or recombinant proteins (AKT and ERK). Each curve was plotted as a function of signal intensity vs. log concentration derived units, CU (Computed Unit). The data were fit to a five parameter equation by nonlinear regression, simultaneously fitting all three dilutions of the capture Ab. Fitting was carried out using R, an open source statistical software package (<http://www.r-project.org/>). The individual predictions from each of the standard curves (3 capture Ab dilutions and 4 PMT gain-set scanning) were combined into a single, final prediction. The final prediction was calculated by a weighted (determined by the slope) average of the individual predictions and then designated as CU [[17-19](#_ENREF_17)]. CU is a representation of marker expression in unknown samples relative to that of control cell lines with known expression levels. Because expression of each marker is determined in unique CEER assays with different cell line standards, only CU values of the same marker across various samples can be compared.
